# Supplementary material for: Content-rich biological network constructed by mining PubMed abstracts
Source: BMC Bioinformatics. 2004 Oct 8;5:147. doi: 10.1186/1471-2105-5-147 (PMC528731; doi:10.1186/1471-2105-5-147)
Supplement: Additional File 2 — The original results of the above study (non-essential files are deleted to keep the file size under the limit set by BMC bioinformatics). [file 1471-2105-5-147-S2.bz2 › chilibotAdditionalFile2/dip05/534ID9238003E10878/html/AR_GTF2F1.html]

 


 **AR** and **GTF2F1** 
  
Found 4 abstracts in PubMed, retrieved 4.  
 

 What does Google say? 
 PDF only 
| .edu only 

---

**Interactive relationship** (e.g. stimulation, inhibition, etc)

**Neutral relationship**- Taken together these results suggest that one mechanism of transcriptional activation by the  **AR**  involves binding to TFIIF  [ **GTF2F1** ]  and recruitment of the transcriptional machinery.  Ref: 9238003 Proc Natl Acad Sci U S A, 1997
- Competition experiments demonstrated that the  **AR**  could interact with the holo TFIIF  [ **GTF2F1** ]  protein.  Ref: 12181312 J Biol Chem, 2002
- Previously we identified an interaction between the  **AR**  transactivation domain amino acids 142 485 and the general transcription factor TFIIF  [ **GTF2F1** ]  McEwan, I.  Ref: 12181312 J Biol Chem, 2002
- The androgen receptor  [ **AR** ]  interacts with multiple regions of the large subunit of general transcription factor TFIIF  [ **GTF2F1** ] .  Ref: 12181312 J Biol Chem, 2002
- Both the amino terminal 136 amino acids and the carboxyl terminal 155 amino acids of RAP74  [ **GTF2F1** ]  interacted with the  **AR**  transactivation domain and were able to rescue basal transcription after squelching by the  **AR**  polypeptide.  Ref: 12181312 J Biol Chem, 2002
- In a protein protein interaction assay the  **AR**  N terminus amino acids 142 485 selectively bound to the basal transcription factors TFIIF  [ **GTF2F1** ]  and the TATA box binding protein TBP.  Ref: 9238003 Proc Natl Acad Sci U S A, 1997
- Interaction of the human androgen receptor  [ **AR** ]  transactivation function with the general transcription factor TFIIF  [ **GTF2F1** ] .  Ref: 9238003 Proc Natl Acad Sci U S A, 1997
- Point mutations within  **AR**  transactivation domain distinguished the binding sites for RAP74  [ **GTF2F1** ]  and the p160 coactivator SRC 1a and identified a single copy of a six amino acid repeat motif as being important for RAP74  [ **GTF2F1** ]  binding.  Ref: 12181312 J Biol Chem, 2002
- Analyses of  **AR**  mediated transcription suggest that the orchestrated interaction of  **AR**  with the transcription factors IIF TFIIF  [ **GTF2F1** ]  and IIH TFIIH, and positive transcription elongation factor b P TEFb, may increase efficiency of transcriptional elongation from the androgen target genes, such as prostate specific antigen PSA.  Ref: 12648523 J Steroid Biochem Mol Biol, 2003

**Non-interactive relationship** (e.g. studied together, co-existance, homology, etc.)

- We have now mapped the binding sites for the  **AR**  transactivation domain within the RAP74  [ **GTF2F1** ]  subunit of TFIIF  [ **GTF2F1** ] .  Ref: 12181312 J Biol Chem, 2002
